# Differentiation of rabbit bone marrow mesenchymal stem cells into corneal epithelial cells in vivo and ex vivo

**Published:** 2009-01-16

**Authors:** Shaofeng Gu, Chengzhong Xing, Jingyi Han, Mark O.M. Tso, Jing Hong

**Affiliations:** 1 China Medical University, Department of Ophthalmology, The First Affiliated Hospital, Shenyang, China; 2 China Medical University, Department of Oncology, The First Affiliated Hospital, Shenyang, China; 3 Peking University Third Hospital, Peking University Eye Center, Beijing, China; 4 The Wilmer Eye Institute, Johns Hopkins University School of Medicine, Baltimore, MD

## Abstract

**Purpose:**

To examine whether bone marrow mesenchymal stem cells (MSCs) could be differentiated into corneal epithelial cells in vivo and ex vivo.

**Methods:**

In vivo, BrdU labeled rabbit MSCs (Rb-MSCs) were suspended in the fibrin gels and transplanted onto the surface of the damaged rabbit corneas. Histology and molecular phenotype were studied on postoperative day 28. In vitro, labeled Rb-MSCs were cultured for three days in two different systems: (1) Group A: Rb-MSCs were co-cultured with rabbit limbal stem cells (Rb-LSCs) by the Transwell culture system. A suspension of Rb-LSCs was added to the upper membrane surface, and the inserts were positioned in the culture wells, which were incubated with Rb-MSCs; (2) Group B: Supernatant medium that had first been used to culture Rb-LSCs and then filtered with a 0.45 μm filter was used to culture Rb-MSCs. For both groups, immunofluorescence and flow cytometric analysis were used to examine the expression of cytokeratin 3 (CK3) in differentiated Rb-MSCs.

**Results:**

In vivo, the data showed that following transplantation of Rb-MSCs, the rabbit’s damaged corneal surface was successfully reconstructed and that some Rb-MSCs participated in the healing of the injured corneal epithelium and expressed CK3. In vitro, the data showed that Rb-MSCs rapidly differentiated into cells with a morphological and molecular phenotype of corneal epithelial-like cells. For both groups, the differentiated Rb-MSCs were positive for corneal epithelial-specific marker CK3. In Group A, flow cytometry analysis showed that at day one, only 3.46±1.9% of cells expressed CK3. This increased to 7.24±3.80% at day two and decreased slightly (5.50±3.33%) at day three. The proportion of CK3 in Group B was 4.09±1.84% at day one, rising to 9.31±5.92% after 24 h, but falling (4.37±2.61%) at day three. The mean differences are significant between each group and the negative control, but was not significant between Group A and Group B.

**Conclusions:**

MSCs could differentiate into corneal epithelial-like cells in vivo and ex vivo.

## Introduction

The corneal epithelium is a rapidly regenerating stratified epithelium which plays a critical role in maintaining corneal transparency and integrity of the ocular surface. The maintenance of the corneal epithelial cell mass is achieved by limbal stem cells (LSCs) located in the basal epithelium of the limbus [1]. The partial or total deficiency of the LSCs population may greatly affect ocular surface integrity and stability. After almost 20 years of studies, there are still no reliable therapies for severe forms of LSCs dysfunction, especially when LSCs deficiencies are bilateral. It is important to test the suitability of other stem cell lines for the reconstruction of a stem cell-deficient ocular surface.

Due to their great expansion and differentiation potential, particular attention has been focused on mesenchymal stem cells (MSCs). MSCs are a self-renewing, multipotent stem cell group that mainly present in bone marrow (BM) [2,3]. Numerous reports have indicated that MSCs can give rise to a broad spectrum of tissues, including bone, cartilage, adipose tissue, and muscular tissue both in vivo and ex vivo [4,5]. In response to appropriate experimental conditions, MSCs could also differentiate into cells of all three germ layers [6,7]. This type of cross-lineage differentiation is known as transdifferentiation, which implies that adult stem cells contain multidifferentiation potentials. It has been shown in vivo that MSCs can generate epithelial cell types in skin, lung, and other tissues [8-10]. One study suggested that systemically transplanted MSCs can engraft to an injured cornea and promote wound healing by differentiation, proliferation, and synergizing with hematopoietic stem cells [11]. A recent report suggested that transplantation of human mesenchymal stem cells (hMSCs) could reconstruct the damaged cornea. The therapeutic effect of the transplantation may be associated with the inhibition of inflammation and angiogenesis [12]. Data are rare, however, about the feasibility that MSCs could differentiate into corneal epithelial cells, and there is still great controversy concerning MSCs plasticity [13,14]. In this study, we examined whether MSCs can differentiate into corneal epithelial cells in vivo and ex vivo.

## Methods

### Animals

Adult New Zealand white rabbits (1.5–2 kg) were purchased from Peking University Animals Science Research Center. All animals were housed and treated in accordance with the ARVO Statement for the Use of Animals in Ophthalmic and Vision Research.

### Materials

Dulbecco’s modified Eagle’s medium (DMEM), Dulbecco’s modified Eagle’s medium/Ham’s F-12 nutrient mixture (1:1; DMEM/F-12), and Dispase II were purchased from Invitrogen-Gibco BRL (Invitrogen, Grand Island , NY). Fetal bovine serum (FBS) was purchased from Hyclone (Hyclone, Logan , UT). Human epidermal growth factor (hEGF) was purchased from PeproTech Inc. (PeproTech, Rocky Hill , NJ). Dimethyl sulfoxide (DMSO), insulin-transferrin-sodium selenite media supplement, Human type VI collagen, hydrocortisone, Percoll, 5-bromo-2-deoxyuridine (BrdU), anti-BrdU antibody, and FITC- and Cy3-conjugated secondary antibodies were purchased from Sigma-Aldrich (Sigma-Aldrich, St. Louis , MO). The primary antibodies against cytokeratin3 (AE5 clone), and integrin β1 were purchased from Chemicon International, Inc. (Chemicon, Temecula , CA). Anti-P63 monoclonal antibody and CD34 antibody were purchased from Neomarkers (Lab Vision Corporation, Fremont , CA). All plastic cell culture wares were from Corning Costar Co. (Corning Inc, Lowell , MA). Culture plate inserts used to construct the culture system were purchased from Millipore Co. (Millipore, Billerica , MA). Tissue-Tek OCT was purchased from Electron Microscopy Sciences (Electron Microscopy Sciences, Fort Washington , PA). The fibrin gel was provided by Professor Bin Han (Chief scientist of Yiling Biological Corporation kindly presented fibrin gel to us).

### Model of corneal damage

New Zealand white rabbits (n=20) were subjected to corneal damage in the right eye as previously described [15]. All procedures were performed under general anesthesia (2 mg/kg ketoprofen and 1 mg/kg acepromazine given subcutaneously). Corneal wounds were made in the right eye of each rabbit by contact with filter paper (15 mm diameter; 30 s contact time) soaked with 1 N NaOH and then washed with 0.9% NaCl. Antibiotic drops were applied to the wounded eye three times per day.

### Isolation and culture of rabbit LSCs and MSCs

Rb-LSCs used for cell-suspension culture were isolated from New Zealand white rabbit limbal tissues by modification of a method previously described [16]. Rabbit limbal tissues were washed in PBS containing 100 U/ml penicillin and 50 μg/ml gentamicin. After carefully removing the corneal endothelium, iris, excessive sclera, conjunctiva, and subconjunctival tissue, the limbal rings were exposed to dispase II (1.2 IU/ml in Hanks’ balanced salt solution free of Mg2^+^ and Ca2^+^) at 37 °C for 1 h. The loosened epithelial sheets were removed with a cell scraper and separated into single cells by 0.25% trypsin + 0.02% EDTA for 10 min at 37 °C. Cells were pelleted at 1,000 rpm for 5 min and resuspended in SHEM. SHEM consisted of an equal volume of Dulbecco’s modified Eagle’s medium (DMEM) and Ham’s F12, supplemented with 5% fetal bovine serum, 5 μg/ml insulin, 5 μg/ml transferrin, 5 μg/ml sodium selenite, 10 ng/ml epidermal growth factor, 0.5% dimethyl sulfoxide, 0.5 μg/ml hydrocortisone, and 100 IU/ml penicillin-streptomycin. Cells were plated at 10^4^ cells/cm^2^ in cell culture dishes containing MMC-treated 3T3 feeder layer and incubated at 37 °C. Immunofluorescence was used to check the molecular phenotype of Rb-LSCs.

Rb-MSCs were obtained from the femurs of adult New Zealand white rabbits as previously described [17]. In brief, 10 ml bone marrow was diluted 1:2 with phosphate buffer solution (PBS) and loaded over 5 ml Percoll (density, 1.077). Cells were harvested from the interface after centrifugation at 2,000 rpm for 20 min and washed with DMEM. Cells were resuspended in DMEM containing 10% fetal bovine serum, 100U/ml penicillin, 100 g/ml streptomycin, and incubated at 37 °C. Flow cytometric analysis was used to check the molecular phenotype of Rb-MSCs.

### In vitro adipogenesis evaluation of Rb-MSCs

For adipogenic differentiation assays, Rb-MSCs were plated to a 6 well plate (5,000/cm^2^) and grown to 80% confluence. The adipogenic differentiation was induced by using Dulbecco’s modified Eagle medium (DMEM) supplemented with 10% FBS, 0.5 mM isobutylmethylxanthine, 10 μM insulin, 1 μM dexamethasone, and 200 μM indomethacin for 2 weeks with full medium changes performed every 3 days. To detect fat deposition in the cells, cells were fixed with 4% paraformaldehyde for 30 min at room temperature and stained with oil red O.

### Labeling Rb-MSCs by BrdU

To obtain cells for later identification expressing 5-bromo-20-deoxyuridine (BrdU), media containing 10 μmol/l BrdU was added to 70% confluent cultures for 24 h. To investigate its efficiency for cell labeling, at the completion of the incubation period, the cells were stained with antibodies against BrdU. Cultured cells on chamber slides were fixed with ice-cold acetone/methanol (1:1) at 20 °C for 30 min. After incubating with 2 N of HCl at 20 °C for 30 min and washed three times in PBS, the slides were incubated overnight with primary antibodies against BrdU at a 1:500 dilution at 4 °C. Negative control samples were incubated in PBS without the primary antibodies. After incubation with the proper secondary antibody at 37 °C for 45 min, slides were mounted with anti-fading mounting medium and examined using fluorescence microscope equipment (Olympus BX51, Olympus Co., Ltd., Tokyo, Japan).

### Transplantation of grafts with (or without) Rb-MSCs

Twenty eight days after the corneal injury, fibrin gels were transplanted onto the rabbit cornea. For the transplantation, the rabbits with damaged cornea were divided into two random groups. Group 1 underwent a graft of fibrin gel with labeled Rb-MSCs (the labeled Rb-MSCs were suspended in a fibrin gel). For Group 2, the transplanted to fibrin gel did not contain Rb-MSCs. For all the rabbits, the damaged corneal surface was carefully keratectomized under anesthesia. The surgery procedures were the same in both groups. A 360° limbal peritomy was performed with Vanass scissors and Bonn forceps. The corneal epithelium that remained was scraped off from the central cornea with a number 15 scalpel blade. The fibrin gel with (or without) Rb-MSCs was placed on the cornea with the conjunctiva covering the edges. The grafts were secured in place by four interrupted cardinal sutures of 10–0 nylon. To protect the fresh graft, a complete central tarsorrhaphy was achieved with a single 5–0 mattress suture. Seven days after surgery, the eyelid was opened. After the eyelid was opened, antibiotic drops were applied to the operated eye three times a day throughout 21 day.

Rabbit corneas were observed with a slit lamp microscope (SL-1600; Nidek Co., Ltd., Aithi, Japan) every day to evaluate reepithelialization, neovascularization, and transparency. On postoperative day 28, rabbits were euthanized with a 10% overdose of trichloroacetaldehyde monohydrate solution for histology and immunofluorescence study.

### Differentiation protocols in vitro

To induce differentiation in vitro, BrdU labeled Rb-MSCs were divided into two groups: (1) Group A: Rb-MSCs were established using 0.45 μm MILLICELL-HA membrane Transwell culture system. A suspension of Rb-LSCs was added to the upper membrane surface and inserts were positioned in the culture wells, which had been incubated with Rb-MSCs. (2) Group B: Rb-MSCs were cultured with supernatant medium. The supernatant medium used to culture Rb-MSCs had first been used to culture Rb-LSCs and were then filtered with a 0.45 μm filter. After induced treatment, the expression of cytokeratin 3 (CK3) was observed by immunofluorescence and flow cytometric analysis. The process was repeated three times in all groups. Pure Rb-MSCs were used as negative controls.

### Histology and Immunofluorescence

At the end of the experiment, rabbit corneas from the in vivo experiments were collected, fixed in 4% paraformaldehyde, and embedded in Tissue Tek OCT compound for sectioning. The cells grown in glass chamber slides were fixed with ice-cold acetone/methanol (1:1) for 30 min at room temperature. For hematoxylin and eosin (H&E) staining, consecutive sections (6 μm) were routinely washed, stained, and observed with a light microscope (Olympus BX51; Olympus Co., Ltd., Tokyo, Japan). For immunofluorescence, the cells and the sections were incubated with 3% BSA/0.1% Triton X-100/PBS for 30 min at room temperature to block unspecific binding sites. The cells were incubated with primary antibody (CK3 1:100, p63 1:50, integrinß1 1:50), and the sections were incubated overnight with anti-CK3 (1:100) antibody at 4 °C. After washing three times in PBS, the slides were incubated with proper secondary antibody at 37 °C for 45 min. Isotype-matched primary antibodies served as controls. Specimens were mounted with anti-fading mounting medium and examined using fluorescence microscope equipment (Olympus BX51; Olympus Co., Ltd.).

Double staining with CK3 and BrdU was performed on cells grown on coverslips and on the sections. After incubation with antibody directed against CK3 (1:100) and Cy3-conjugated secondary antibody, the slides were subjected to anti-BrdU (1:100) antibody and FITC-conjugated secondary antibody. After covering with anti-fading mounting medium, the specimens were examined by confocal microscopy (Zeiss 510 META; Carl Zeiss Co, Ltd, Jena , Germany).

### Flow cytometric analysis

Cells were harvested by trypsinization, and nonspecific binding was prevented by incubating the cells in 3% BSA for 30 min. Approximately 5x10^5^ cells were suspended in a final volume of 100 μl of flow cytometric (FC) buffer and incubated with the primary antibody (CK3 1:100, CD34 1:100, and CD29 1:100) or isotype contro1 at 4 °C for 30 min. After being washed twice, the cells were incubated with proper secondary antibody in FC buffer for 30 min at 4 °C. The cells were washed twice with cold FC buffer and suspended in a final volume of 500 μl for flow cytometric analysis. Fluorescent cell analysis was performed with FACSC alibur cytometer (Becton Dickinson Immunocytometry System, San Jose, CA) and data analyzed by FlowJo software (Tree Star, Inc., Ashland, OR).

### Statistical Analysis

Results are expressed as the mean±SD% from three replicate experiments. Where appropriate, differences were analyzed by ANOVA. A p<0.05 was regarded as statistically significant.

## Results

### Morphology and phenotype of Rb-LSCs and Rb-MSCs

**Figure 1:**
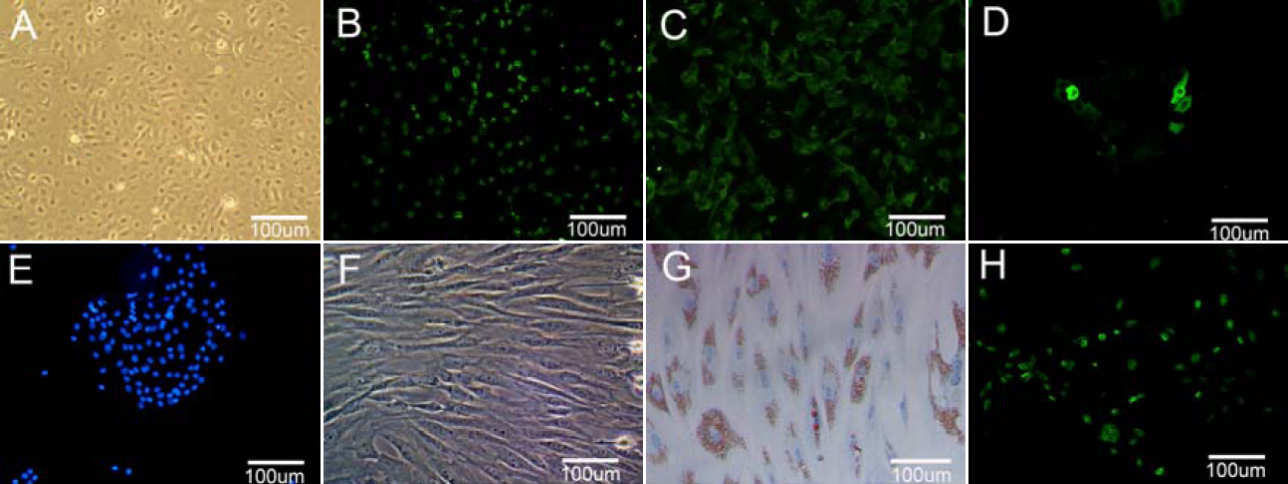
Characterization of Rb-LSCs and Rb-MSCs in vitro. A light photomicrograph of cultured Rb-LSCs is seen in panel **A** (200X). Positive staining of p63 (**B**) and integrinβ1(**C**) were observed on Rb-LSCs (200X). CK3 was positive on few of Rb-LSCs (**D**) and Hoechst 33342 (**E**) was used as a counterstain (200X). A light photomicrograph of cultured Rb-MSCs is seen in panel **F** (200×). Under the adipogenic induction medium cultured for 14 days, Rb-MSCs showed a positive reaction with oil red O stain (**G**) (200X). **H**: Immunofluorescent staining showing Rb-MSCs labeled by BrdU expressed green fluorescence in the cell nucleus (200X).

**Figure 2:**
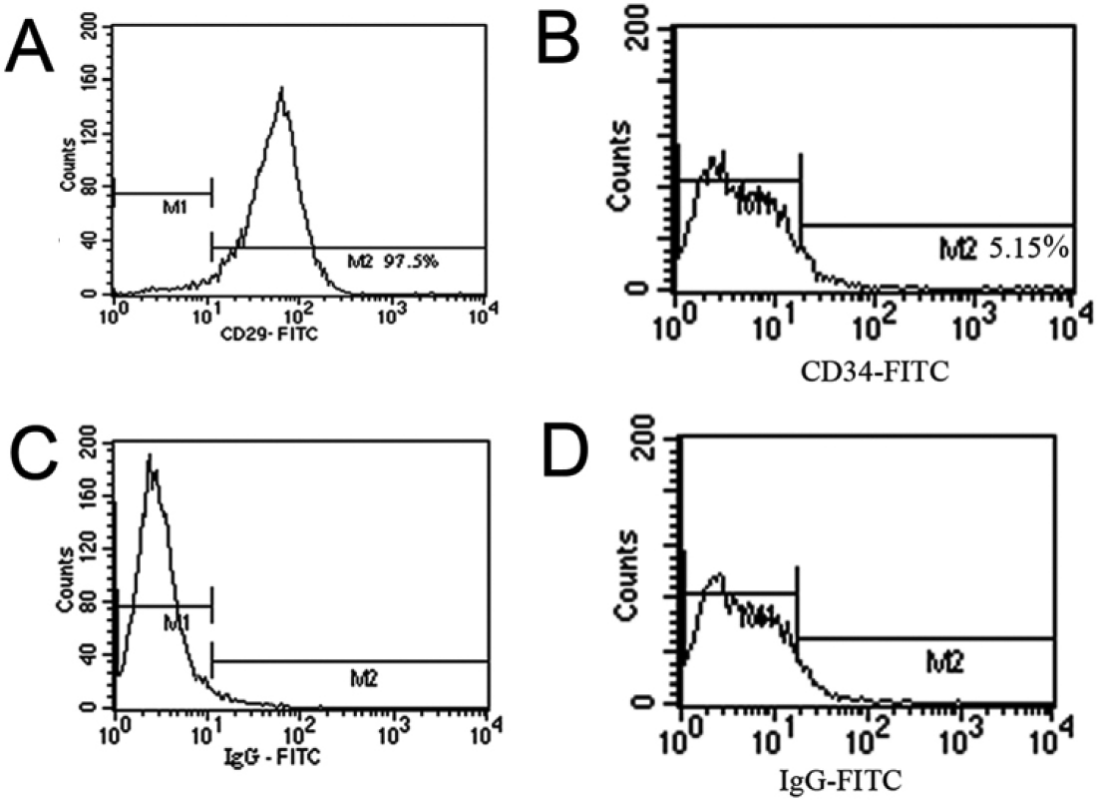
Flow cytometric analysis of the Rb-MSCs. The data showed that 97.5% of cells were positive for CD29 (**A**) and 5.15% of cells were positive for CD34 (**B**). **C** and **D**: Negative controls.

Within 24 h after seeding, most Rb-LSCs attached to plastic and started spreading. Small colonies, including approximately 8–10 cells, were formed day 3–4 after inoculation and extended very quickly thereafter. The cell morphology appeared to be compact, uniform, and small polygon in shape. By day 10–14, colonies began to fuse and formed a monolayer on confluence (Figure 1A). Immunofluorescence revealed that most Rb-LSCs were positive for p63 (Figure 1B), integrinß1 (Figure 1C), and some of the cultured cells expressed cytokeratin 3 (Figure 1D,E). MSCs were successfully established from bone marrow collected from 10 anesthetized rabbits (n=10). Most of the non-adherent cells were removed during the first media change at 24 h. Colonies of fibroblast-like cells attached to the plastic were evident at day 4–5 after initial seeding. Most cell lines were composed of cells with a characteristic spindle shape. The number and size of the colonies reached 80% confluence by day 14–15 (Figure 1F). After adipogenic induction, intracytoplasmic lipid vesicles were observed by oil red O staining in induced Rb-MSCs (Figure 1G). Immunofluorescence revealed that Rb-MSCs labeling by BrdU expressed green fluorescence in the cell nucleus (Figure 1H). Flow cytometry analyses confirmed that 97. 5% of the cells expressed CD29 antigen and 5.15% of the cells expressed CD34 antigen (Figure 2).

### Differentiated analysis of Rb-MSCs in vivo

#### Preparation of Graft

**Figure 3:**
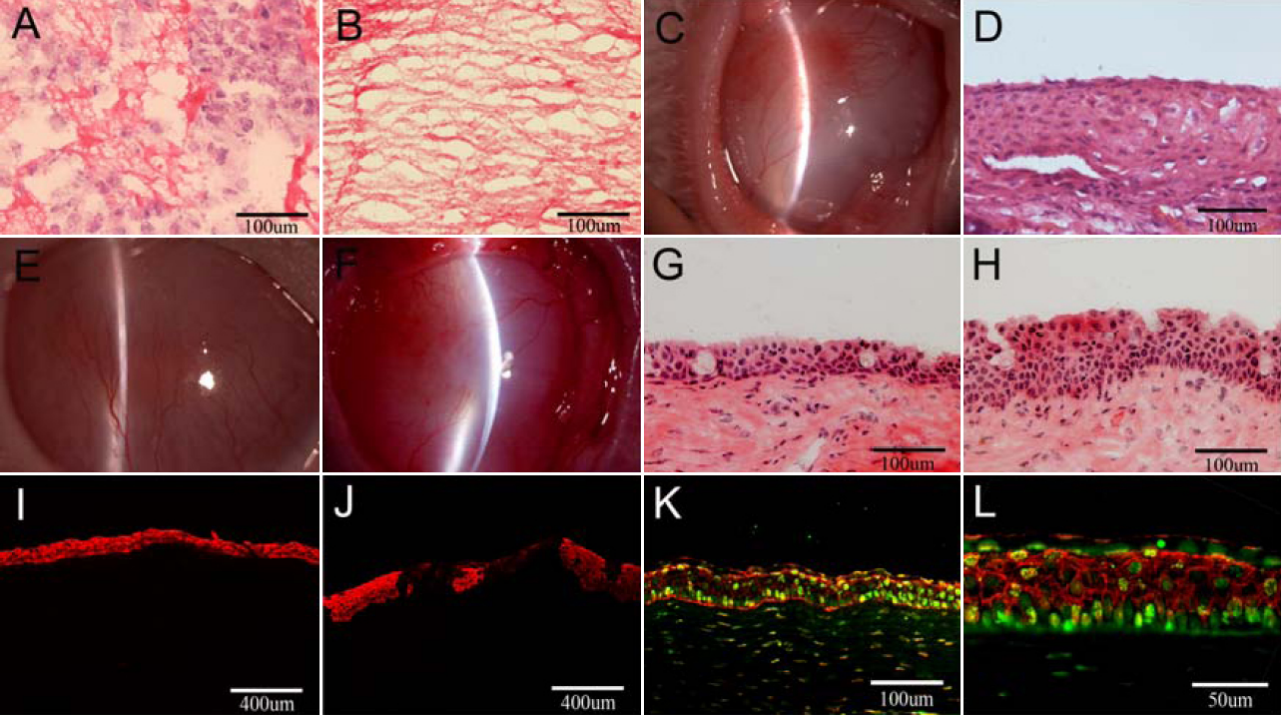
Characterization of the fibrin gel and the rabbit corneas after transplantation. Hematoxylin and eosin staining of Group 1 (**A**) showed the fibrin gel with Rb-MSCs and Group 2 (**B**) the fibrin gel without cells (400X). A slit-lamp photograph (**C**) and hematoxylin and eosin staining (**D**) showed the model of limbal stem cell deficiency (400X). A slit-lamp photograph of the rabbit corneas after transplantation showed the opacification and neovascularization in Group 1 (**E**) and in Group 2 (**F**). Hematoxylin and eosin staining showed that goblet cells, new vessels, and inflammatory cells were present in some regions in Group 1 (**G**) and Group 2 (**H**; 400X). Positive CK3 staining was continuous throughout whole corneal epithelium in Group 1 (**I**), but was irregular in Group 2 (**J**) by immunofluorescent staining (100X). Double staining showed corneal epithelial cells expressed BrdU (green) and CK3 (red) in Group 1 (**K**; 200X). A higher magnification of the double staining is seen in panel **L** (400X).

The fibrin gel was semi-transparent. Transverse sectioning of Group 1 revealed that labeled Rb-MSCs penetrated into the pores of fibrin gel by H&E staining (Figure 3A). The fibrin gel of Group 2 did not show cells by H&E staining (Figure 3B).

#### Model of corneal damage

Limbal stem cell deficiency was achieved at day 28. Analysis by slit lamp photography showed thickened corneas, stromal scarring, and subepithelial neovascularization (Figure 3C). When compared to the non-wounded cornea, histological staining showed that the rabbit conjunctival epithelium completely covered the damaged corneal surface with variable cell layers and the new vessels infiltrated the epithelium. (Figure 3D).

#### Observation of Graft

Analysis by slit lamp photography showed that the appearance of the lids, cornea, and conjunctiva improved with time. Corneal epithelium was epithelialized completely in both groups at day 28 after surgery, but neovascularization and corneal opacification presented in both groups within the four-week period after surgery (Figure 3E,F). There was no difference of cornea opacity (χ^2^=0.267, p=0.606) and neovascularization (χ^2^=2.222, p=0.136) between Groups 1 and 2 (Table 1). Histological staining showed that goblet cells, new vessels and inflammatory cells presented in some region in both groups (Figure 3G,H). To confirm and characterize these epithelial cells, we investigated the expression of CK3 by immunofluorescent staining. As shown in Figure 3, expression of CK3 was observed in both groups. However, positive CK3 staining was continuous throughout the whole corneal epithelium in Group 1 (Figure 3I), but was irregular in Group 2 (Figure 3J). To examine the nature of the epithelial cells covering the damaged cornea, we studied the expression of BrdU and CK3 by confocal microscopy. In Group 1, the data revealed that corneal epithelial cells in some regions expressed CK3 alone, while other regions expressed both BrdU and CK3 (Figure 3K,L). In Group 2, the expression of BrdU was not detected.

**Table 1:** Clinical observation of the damaged corneal surface at day 28 following transplantation.

**Variable**	**Group 1 (n=10)**	**Group 2 (n=10)**
**Corneal transparency**		
Completely transparent	0 (0%)	0 (0%)
Iris was clear (partially obscure)	3 (30%)	2 (20%)
Iris was not clear (completely obscure)	7 (70%)	8 (80%)
**Neovascularization**		
No neovascularization	0 (0%)	0 (0%)
Detected within 3 mm from the limbus	2 (20%)	0 (0%)
Detected over 3 mm from the limbus	8 (80%)	10 (10%)

Data was evaluated based on the transparency (χ^2^=0.267, p=0.606) and neovascularization (χ^2^=2.222, p=0.136).

### Differentiated analysis of Rb-MSCs in vitro

#### Morphological and molecular phenotype of differentiated Rb-MSCs in vitro

**Figure 4:**
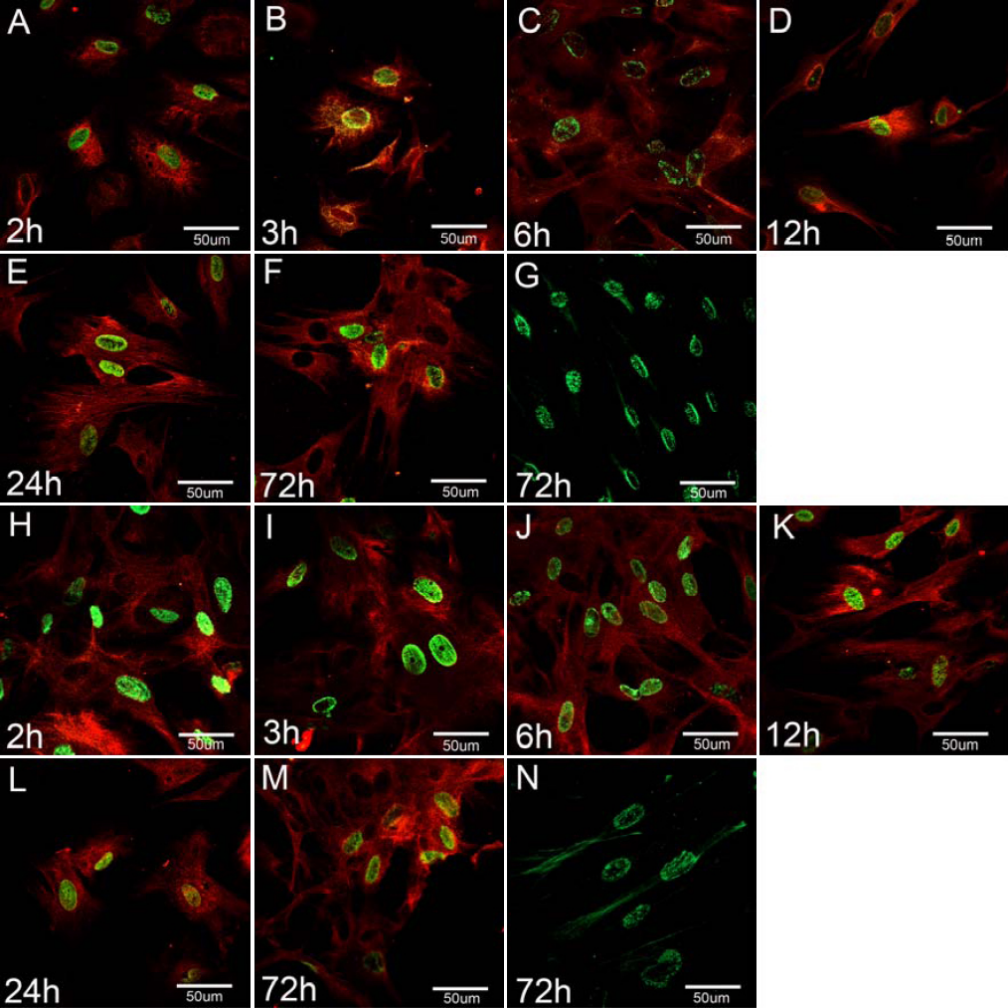
Morphological and phenotypic of differentiated Rb-MSCs in vitro. Double staining showed expression of CK3 and BrdU both in Group A (**A**-**F**) and Group B (**H**-**M**) as of day three. Only BrdU positive staining showed in negative controls (**G**,**N**). The red staining was CK3 and the green color was BrdU (400X).

In vitro, to further test the hypothesis that Rb-MSCs are capable of differentiation into corneal epithelial cells, we cultured Rb-MSCs with two different induction methods. In all groups we found that a subset of the adherent Rb-MSCs rapidly lose their characteristic fibroblast morphology, becoming broad and flattened with an epithelial shape. To determine the molecular phenotype of differentiated Rb-MSCs, the expression of CK3 (special proteins of corneal epithelial cells) was examined by confocal microscopy. CK3 positive Rb-MSCs were first observed within 2 h and there was little difference between different groups. Multinucleated Rb-MSCs were not found. The morphological and phenotypic differentiated Rb-MSCs remained unchanged in both groups as of day three (Figure 4).

#### Differentiated ratio analysis of different groups in vitro

**Figure 5:**
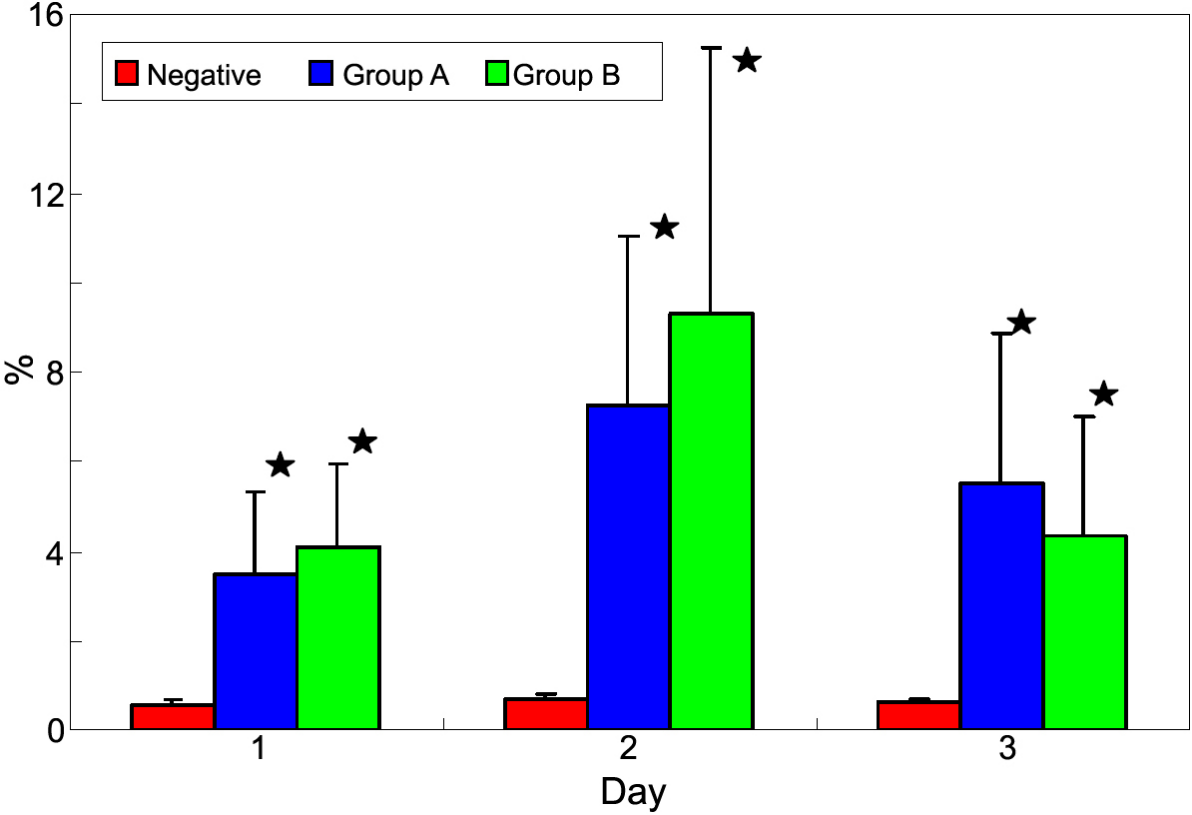
Flow cytometry analysis of the ratio of CK3+ cells in all groups. Data represent the mean±SE% of results in three replicate experiments (The asterisk indicated a p*<*0.05, when compared with control data). There were no significant differences between Group A and Group B (p*>*0.05).

To determine the extent of differentiation, flow cytometry analyses (FCAS) were used to assay the total population of differentiated Rb-MSCs. In Group A, FCAS showed that only 3.46±1.90% of cells expressed CK3 at day one, increasing to 7.24±3.80% at day two, and slightly decreasing (5.50±3.33%) at day three. In Group B, the proportion of CK3 was 4.09±1.84% at day one, rising to 9.31±5.92% after 24 h, but falling significantly (4.37±2.61%) at day three. The proportion of CK3+ cells remained steady in the negative group (0.78±0.1% at day 1, 0.66±0.15% at day 2, and 0.55±0.11% at day 3). The mean difference is significant between each group and negative control (p<0.05), but is not significant between Group A and Group B (p>0.05; Figure 5).

## Discussion

Bone marrow contains hematopoietic stem cells (HSCs) which produce all the blood cells and mesenchymal stem cells (MSCs) differentiate into mesenchymal lineages. It has been observed that MSCs express several surface markers, such as CD29, CD90, and CD105, but do not express the hematopoietic markers CD34 and CD45 [18]. In this study, from rabbit bone marrow, we successfully isolated MSCs with a characteristic spindle shape and cultured Rb-MSCs that could be induced into adipose cells. Flow cytometry analysis revealed that most cells expressed the mesenchymal marker CD29 and did not express CD34, a hematopoietic marker. Morphology, surface antigen profile characteristics, and differentiation characteristics provided evidence that the cells isolated in our study were MSCs.

MSCs are pluripotent precursor cells, which can differentiate into mesenchymal cells and also into cell tissue types other than their own lineage in vivo and ex vivo [19]. It has been suggested that there are two general strategies that regulate stem cell fate; intrinsic and extrinsic signals. The extrinsic signals that control stem cell fate and collectively make up the stem cell microenvironment or niche, include: secreted factors, cell-cell interactions mediated by integral membrane proteins, and integrins and extracellular matrix [20]. We hypothesized that in response to appropriate conditions, MSC could differentiate into corneal epithelial cells. To examine the hypothesis, we developed an in vivo model of corneal epithelial damage and repaired it by fibrin gel with or without labeling Rb-MSCs. We found that the corneal epithelium was reconstructed completely in both groups, but that there was no statistical difference of cornea opacity and neovascularization between Group 1 and Group 2 at day 28 after surgery. We differed from the study of Ma et al. [12] in which they reported that the transplantation of MSCs could repair the damaged cornea whereas transplantion without MSCs could not. We believe that this result can be explained by the difference of species, the degree of corneal damage, and transplant methodology.

To confirm the characterization of these epithelial cells, we investigated the expression of CK3 and found positive CK3 staining presented in both groups. In Group 1, the expression of CK3 was continuous throughout the entire corneal epithelium, but was irregular in Group 2. This signifies that there are epithelial-like cells covering the damaged rabbit cornea. To examine the nature of these cells, we studied the expression of BrdU and CK3 by double staining. The results revealed the co-expression of BrdU and CK3 in Group 1. This means that in Group 1, some Rb-MSCs participated in the healing of injured corneal epithelium and might differentiate into corneal epithelial-like cells in vivo. However, CK3+ cells were also found in Group 2. We believe this result can be explained by the works of Kurpakus et al. [21] who demonstrated that CK3 expression is inactivated in the conjunctiva but not when conjunctival cells are maintained in a corneal environment.This result suggested that Rb-MSCs may differentiate into corneal epithelial cells in vivo.

To examine whether Rb-MSCs can differentiate into corneal epithelial cells in vitro, we co-cultured BrdU labeled Rb-MSCs with Rb-LSCs in a Transwell culture system, which permits only chemical factors to diffuse but not cells (Group A). The results showed a subset of Rb-MSCs rapidly differentiated into corneal epithelial cells and expressed CK3 as of day three. As a Transwell culture system does not allow direct cell-to-cell contact, soluble chemical factors in the culture may be a cause of Rb-MSCs’ differentiation. We hypothesized that factors secreted by Rb-LSCs may play an important role in Rb-MSCs corneal differentiation. To test this hypothesis, we cultured Rb-MSCs in conditioned medium which had been used to culture Rb-LSCs and filtered with 0.45 um filter (Group B). Double staining showed the expression of CK3 to be the same as Group A. FACS analyses showed expression of CK3 in differentiated Rb-MSCs was similar in both groups. Statistical Analysis showed the mean difference is significant between each group and negative control (p<0.05), but is not significant between Group A and Group B (p>0.05). In Group A, Rb-MSCs were cultured in a microenvironment which permits only secreted chemical factors to diffuse. In Group B, cells cultured in conditioned medium were affected by some chemical factors, which had already been secreted from Rb-LSCs. For both groups, we did not co-culture Rb-MSCs with Rb-LSCs in direct cell-to-cell contact, which demonstrated that Rb-MSCs could differentiate into corneal epithelial cells in vitro and indicated that some chemical factors secreted by Rb-LSCs may play an important role in this process.

In vitro, with immunofluorescent staining and FACS analyses, we noted that as of day three, the morphology and phenotype of differentiated Rb-MSCs remained unchanged in both groups. We considered that this was due to not enough time having yet elapsed to observe an obvious change. At the same time, FACS analyses showed that the level of CK3 decreased in day three for all in vitro groups. We believed cell apoptosis might be the reason for this decrease. The factors which induced differentiation might also have participated in the cell apoptosis.

Although it was observed that Rb-MSCs differentiated into corneal epithelial-like cells, it should be noted that this study only demonstrated short-term results. Moreover, Rb-MSCs are multipotent stem cells whereas corneal epithelial cells are terminal differentiated cells and do not belong to the same germ layer. The precise process of this differentiated reprogramming remains to be determined. Future studies will be needed to determine if a key switch contributes to this process, and the key transcription signals which regulate this process. Despite its preliminary character, our study demonstrated that Rb-MSCs participated in the healing of injured corneal epithelium and could be induced into corneal epithelial-like cells in vivo and ex vivo, providing a new source of cells for the treatment of corneal disorders.
